# Extracellular vesicles in diabetes mellitus induce alterations in endothelial cell morphology and migration

**DOI:** 10.1186/s12967-020-02398-6

**Published:** 2020-06-09

**Authors:** Sharon F. Wu, Nicole Noren Hooten, David W. Freeman, Nicolle A. Mode, Alan B. Zonderman, Michele K. Evans

**Affiliations:** 1 Laboratory of Epidemiology and Population Science, National Institute on Aging, National Institutes of Health, Baltimore, MD 21224 USA; 2 Present Address: University of Utah School of Medicine, Salt Lake City, UT USA

**Keywords:** Extracellular vesicles, Microvesicles, Exosomes, Diabetes mellitus, Inflammation, Endothelial cells, Cell morphology, Cell migration, Proteomics, VEGF-A

## Abstract

**Background:**

Inflammation-related atherosclerotic peripheral vascular disease is a major end organ complication of diabetes mellitus that results in devastating morbidity and mortality. Extracellular vesicles (EVs) are nano-sized particles that contain molecular cargo and circulate in the blood. Here, we examined EV protein cargo from diabetic individuals and whether these EVs cause functional changes in endothelial cells.

**Methods:**

We quantified inflammatory protein levels in plasma-derived EVs from a longitudinal cohort of euglycemic and diabetic individuals and used in vitro endothelial cell biological assays to assess the functional effects of these EVs with samples from a cross-sectional cohort.

**Results:**

We found several significant associations between EV inflammatory protein levels and diabetes status. The angiogenic factor, vascular endothelial growth factor A (VEGF-A), was associated with diabetes status in our longitudinal cohort. Those with diabetes mellitus had higher EV VEGF-A levels compared to euglycemic individuals. Additionally, EV levels of VEGF-A were significantly associated with homeostatic model assessment of insulin resistance (HOMA-IR) and β-cell function (HOMA-B). To test whether EVs with different inflammatory cargo can demonstrate different effects on endothelial cells, we performed cell migration and immunofluorescence assays. We observed that EVs from diabetic individuals increased cell lamellipodia formation and migration when compared to EVs from euglycemic individuals.

**Conclusions:**

Higher levels of inflammatory proteins were found in EVs from diabetic individuals. Our data implicate EVs as playing important roles in peripheral vascular disease that occur in individuals with diabetes mellitus and suggest that EVs may serve as an informative diagnostic tool for the disease.

## Background

In the United States, type 2 diabetes mellitus affects nearly 30.3 million people, or 9.4% of the population [1]. Type 2 diabetes is a complex, age-associated metabolic disorder characterized by low-grade chronic inflammation [2], hyperinsulinemia [3], insulin resistance, and β-cell dysfunction. It also increases the risk for numerous vascular-related comorbidities, including end-stage renal [4], cardiovascular [5], cerebrovascular [6], coronary artery [7], peripheral vascular [8], chronic kidney [9], and ophthalmological diseases [10]. The epidemic is reflected in the increase of diabetic individuals with cardiovascular disease (CVD) within the last several decades [11]. CVD is the leading cause of death and takes up the highest proportion of healthcare costs for individuals with diabetes [12, 13]. In addition to the aged population, diabetes also causes disproportionate morbidity and mortality among minority groups in the U.S., including African Americans, Hispanics, and Native Americans [1]. In order to create diagnostic tools and therapies that will benefit a broad spectrum of the population, more knowledge of the mechanisms that drive vascular disease in diabetes is needed.

Many of the cardiovascular diseases associated with diabetes mellitus can be attributed to endothelial dysfunction, which is known to cause atherosclerosis [14, 15]. In diabetes mellitus, endothelial dysfunction occurs when blood vessels do not properly vasodilate due to an imbalance in endothelium-derived factors [16]. Oxidative stress and inflammation contribute to the vascular dysfunction [16]. Furthermore, endothelial dysfunction has been associated with insulin resistance [17] and impaired β-cell function [18]. Yet, finding a way to target inflammation to improve vascular endothelial health remains a challenge in clinical trials [19].

Recent data indicate that extracellular vesicles (EVs), which are membrane-derived particles, facilitate intercellular communication in various disease processes [20, 21]. Ranging in size from ~ 30 to 400 nm, EVs shuttle molecular cargo, including lipids, nucleic acids, and proteins, in a cell-to-cell manner. Based on their mode of biogenesis, EVs can be categorized as (a) exosomes, which are secreted from multivesicular bodies upon fusion with the plasma membrane, (b) microvesicles, which pinch off the extracellular surface of the plasma membrane, and (c) apoptotic bodies, which are shed from the plasma membrane during apoptosis of a cell [22]. More recently, fractions isolated during differential ultracentrifugation have been referred to as small EVs (sEVs) and medium-sized EVs, rather than, respectively, exosomes and microvesicles [23].

EVs isolated from blood samples can potentially lead to new and improved diagnostic tools and therapies [24] for a variety of diseases, including diabetes mellitus [25]. For the past few decades, significant attention has been focused on the role of larger EVs, called microparticles, in type 2 diabetes. A recent meta-analysis of 34 studies showed that when compared to non-diabetic controls, individuals with type 2 diabetes have higher levels of total circulating microparticles as well as microparticles derived from platelets, monocytes and endothelial cells [26]. Recent technological advancements have allowed for analysis of EVs (~ 30–400 nm) smaller than microparticles. According to previous longitudinal and cross-sectional studies from our group, diabetic individuals have higher circulating levels of EVs in their plasma than euglycemic controls [27]. Furthermore, we found that insulin resistance increased EV secretion and that EVs from diabetic individuals increased cytokine secretion in monocytes. These findings agree with other human studies suggesting an important role of EVs in facilitating insulin resistance and inflammation [28, 29].

Through the delivery of molecular cargo, EVs can elicit functional effects on target cells, including pathological effects [30]. Not as extensively explored in diabetes research is the possibility that EVs contribute to endothelial dysfunction and thereby lead to the progression of cardiovascular disease. The idea was examined by a recent study, which found that endothelial dysfunction in non-diabetic mice can be induced by treatment with serum-derived EVs containing arginase 1 from diabetic mice [31].

To study the molecular cargo that may contribute to inflammation, insulin resistance and endothelial dysfunction in diabetes mellitus, here we profiled the inflammatory protein content of EVs in a longitudinal cohort of diabetic and euglycemic humans. We then further explored our data by testing the effects of EVs from diabetic individuals on the actin cytoskeletal structure and migratory behavior of human aortic endothelial cells.

## Materials and methods

### Clinical study participants

Longitudinal and cross-sectional cohorts of euglycemic and diabetic individuals were chosen from the Healthy Aging in Neighborhoods of Diversity across the Life Span (HANDLS) study of the National Institute on Aging (NIA) Intramural Research Program, National Institutes of Health (NIH) [32]. HANDLS is a longitudinal study that follows community-dwelling participants, with the goal to better understand age-associated diseases in relation to race and socioeconomic status. Individuals were categorized as diabetic if they reported having received a diagnosis by a healthcare provider, were taking medication for diabetes mellitus or had a fasting serum glucose of > 125 mg/dL. Collection of plasma samples took place after the individual fasted overnight [33].

Previously, we examined EV characteristics in a longitudinal cohort of individuals [27]. This cohort was matched based on body mass index (BMI) category and included participants who had blood samples that were collected during two visits, with a time gap of approximately 5 years (4.95 ± 0.23). Participants included 19 individuals who were euglycemic at both visits, 19 individuals who were euglycemic on the first visit and diabetic during the second, and 20 individuals who were prediabetic on the first visit and diabetic during the second (Table 1). The cross-sectional cohort was matched for BMI and included nine euglycemic and nine diabetic individuals from one visit (Table 1). BMI was categorized as either underweight/normal (< 25 kg/m^2^), overweight (25 to < 30 kg/m^2^), obese class I (30 to < 35 kg/m^2^), and obese class II/III (≥ 35 kg/m^2^). Homeostatic model assessment (HOMA) of insulin resistance (HOMA-IR) and of β-cell function (HOMA-B) were calculated based on fasting serum glucose and insulin levels [34]. HOMA-IR and HOMA-B were natural log-transformed prior to statistical analysis.

**Table 1:** Clinical characteristics of the longitudinal and cross-sectional cohorts

Characteristics	Longitudinal					Cross-sectional		
	Times	NoDx → NoDx	NoDx → DM	PreDM → DM	*P*	NoDx	DM	*P*
N		19	19	20		9	9	
Age	1	42.5 (8.26)	47.84 (8.58)	47.69 (8.68)	0.095	45.97 (10.83)	53.92 (11.09)	0.143
	2	47.6 (8.75)	52.76 (8.5)	52.21 (8.84)	0.141			
BMI	1	33.1 (5.6)	35.4 (7.66)	35.56 (9.11)	0.54	32.9 (5.51)	32.38 (8.29)	0.877
	2	33.92 (5.48)	36.63 (9.41)	35.44 (8.63)	0.585			
Glucose	1	89.42 (4.14)	93.26 (9.13)	110.55 (6.06)	<0.001	87.78 (5.4)	213.11 (100.3)	0.002
	2	89.58 (6.13)	125.53 (40.68)	131.55 (45.81)	0.001			
LDL	1	132.63 (38.20)	133.05 (31.06)	118.45 (32.13)	0.315	125.89 (21.62)	118.78 (48.06)	0.691
	2	123.53 (33.66)	119.42 (37.89)	119.15 (36.06)	0.915			
HDL	1	50.95 (14.25)	50.05 (11.97)	42.55 (10.21)	0.070	60.89 (10.87)	42.56 (12.92)	0.005
	2	55.84 (11.55)	53.47 (15.21)	44.80 (11.01)	0.022			
Smoker, yes	1	5 (50%)	6 (33.3%)	10 (55.6%)	0.389	4 (44.4%)	4 (44.4%)	> 0.999
	2	5 (35.7%)	8 (50%)	8 (44.4%)	0.783			
Race, white		7 (36.8%)	5 (26.3%)	8 (40%)	0.645	3 (33.3%)	4 (44.4%)	> 0.999
Sex, women		14 (73.7%)	12 (63.2%)	14 (70%)	0.776	7 (77.8%)	7 (77.8%)	> 0.999

Mean and SD are shown for continuous variables, and *n* (%) is shown for the categorical variables. For the longitudinal cohort, continuous variables were analyzed using one-way ANOVA, and for the cross-sectional cohort, the Student’s t-test was used. The categorical variables were analyzed in the longitudinal cohort using χ^2^ goodness-of-fit test, and in the cross-sectional cohort using Fisher’s exact test

### ExoQuick EV isolation

Fasting plasma samples were collected as previously described [33]. For both cohorts, EVs were isolated, as previously reported [27], from 0.5 mL of plasma using ExoQuick Exosome precipitation solution (System Biosciences). ExoQuick offers the most reproducible results as well as the greatest ease of isolation for large human cohorts [33]. Samples isolated from ExoQuick were used in the proteomics experiment.

### Differential ultracentrifugation EV isolation

EVs were isolated from plasma using differential ultracentrifugation. Plasma was added to phosphate-buffered saline (PBS) and then centrifuged at 500 *g* for 10 min, at 2500 *g* for 10 min, and 10,000 *g* for 30 min. For the 10,000 *g* spin, a Beckman Coulter ultracentrifuge was used with a SW 55 Ti rotor (K = 48) to isolate pellets for immunoblotting and electron microscopy and a SW 32 Ti rotor (K = 204) for the rest of the experiments. Samples were then centrifuged at 120,000 *g* for 2 h to isolate pellets for immunoblotting and electron microscopy using a SW 55 Ti rotor (K = 48) and for the other experiments using a SW 32 Ti rotor (K = 204). The pellet in each sample was collected and then resuspended in PBS prior to a final spin at 120,000 *g* for 2 h (SW 55 Ti rotor). Pellets from the 10,000 *g* (10K) and 120,000 *g* (120K) spins were resuspended in sterile PBS. For the experiments that involved a vesicle count, the 10K fraction underwent another spin as previously mentioned [27]. For immunoblotting, the EV pellets were directly lysed in 50 µL of Mammalian Protein Extraction Reagent (M-PER) with phosphatase and protease inhibitors. For electron microscopy, the EV pellets were resuspended in sterile 4-(2-Hydroxyethyl)piperazine-1-ethanesulfonic acid (HEPES) buffer at a physiological pH.

### Immunoblotting

CEM (T lymphoblast) cell lysate and equal amounts of lysed EVs were run on an SDS-PAGE gel and then immunoblotted with known EV protein markers, including Alix (sc-271975; Santa Cruz Biotechnology), Flotillin 1 (ab133497; Abcam), CD81 (EXOAB-CD81A-1; System Biosciences), and EV purity marker GM130 (ab52649; Abcam).

### Electron microscopy

Electron microscopy images were taken by the Johns Hopkins University Neurology Microscopy Core using a Veleta camera (Olympus) as previously described [33]. Grids were viewed on a Libra 120 TEM at 120 kV (Zeiss).

### Nanoparticle tracking analysis

Isolated EVs from differential ultracentrifugation were diluted to 1:50 in sterile PBS. Concentrations and size distributions of the samples were determined using nanoparticle tracking analysis (NTA) on the Nanosight NS500 (Malvern Instruments). At a camera level of 14 and detection level of 3, five videos of 20 s each were recorded for every sample. The NanoSight Software NTA 3.2 Build 3.2.16 was used for analysis. For accuracy, the samples for each cohort or experiment were measured around the same time period, on the same instrument, and by the same operator. Calculation of total EV concentration from plasma was done as reported before [33].

### Multiplex Proximity Extension Assay

Plasma-derived EVs from individuals in the longitudinal cohort were lysed in M-PER, containing protease and phosphatase inhibitors. Protein content was quantified using the Bradford assay and adjusted to the same level across all samples. Equal amounts of protein were used as suggested by Olink^®^ Proteomics and since protein amounts do not always correlate with particle counts in EV preparations [35, 36]. 22 μg of protein in 20 µL (f.c 1.1 μg/μl) of each EV lysate were added to 96-well plates and then analyzed with Olink^®^ Proteomics biomarker Inflammation Panel using Proximity Extension Assay (PEA) technology (Olink^®^ Proteomics). PEA allows for sensitive and specific detection of multiple proteins. Experiments were performed blind of group status. Internal controls were used in each step, including a negative control that accounted for background levels and an interplate control that accounted for different plates. Protein data was normalized to inter- and intra-assay controls and represented as normalized protein expression (NPX) units on a log_2_ scale. In total, 92 proteins were tested using the Olink^®^ Inflammation panel. We found 66 proteins that were detectable in the samples. Out of the 66 proteins, 33 proteins met our threshold for being less than 30% at the lower limit of detection, meaning that each of those proteins was present in more than 70% of all the EV samples. Linear mixed model regression was used to analyze the longitudinal data for the remaining proteins. CCL4, CXCL6, and FGF-21 all showed positively skewed distributions, and hence the data for those proteins were natural log-transformed.

### Endothelial cell culture

Human aortic endothelial cells (HAECs; CC-2535; Lonza) were grown in EBM-2 Endothelial Cell Growth Basal Medium-2 (CC-3156; Lonza) with the EGM-2 Endothelial SingleQuots Kit (CC-4176; Lonza). In preparation for the migration assays, HAECs were incubated in serum-free EBM-2 media for 2 h. HAECs were detached from the plate with trypsin, followed by the addition of neutralization solution and HEPES buffer. The cells were then centrifuged and resuspended in serum-free EBM-2 media prior to experimentation.

### Migration assays

The undersides of Transwell polycarbonate inserts (8.0-μm pore; 3422; Corning) were coated with collagen (30 µg/mL) for 1 h at 37 °C and then blocked with 1% BSA in PBS for 1 h at 37 °C. EVs were isolated using differential ultracentrifugation and the fractions from either the 10K or 120K pellets were used. For each test group, EVs from three individuals from the cross-sectional cohort were pooled and added at a dose of ~ 1.8 × 10^8^ EVs to the bottom chamber of the well containing 500 µL of serum-free EBM-2 media. VEGF-A (0.05 μg) was used as a positive control. Serum-starved HAECs, at dosages of ~ 2.5 × 10^5^ cells for the sEV (120K) assay and ~ 1.2 × 10^5^ cells for the medium-sized (10K) EV assay, were added to the transwells and allowed to migrate for 3 h at 37 °C. Filters were then washed with PBS. The remaining HAECs from the top of the transwells were removed. HAECs were fixed with 3.7% formaldehyde in PBS for 15 min and permeabilized with 0.5% Triton in TBS for 3 min. Cells were washed with PBS and the nuclei were stained with 4′,6-diamidino-2-phenylindole (DAPI; 32670; Sigma), followed by another PBS wash. Filters were then cut from the transwells and mounted on slides with an antifade medium. Images were acquired using a Zeiss Axio Observer D1 Inverted Fluorescence microscope with an AxioCam1Cc1 camera at a 40× objective. DAPI-stained nuclei in each field were counted.

### Immunofluorescence

HAECs (~ 15,000 cells) were plated on collagen-coated glass cover slips in EBM-2 media with 5% exosome-depleted fetal bovine serum (A2720803; Thermo Fisher Scientific) for 24 h. For another 24 h, HAECs were incubated with EVs isolated from the 10K fraction of plasma from an individual in the cross-sectional cohort and a previously reported dose of ~ 1000 vesicles per cell was used [33]. Three replicate experiments of each category, euglycemic and diabetic, were tested. HAECs were washed twice with PBS and fixed in 3.7% formaldehyde/PBS for 15 min, and then permeabilized using 0.5% Triton X-100 for 3 min. HAECs were washed twice in 1X TBS prior to actin-staining with Rhodamine Phalloidin (R415; Thermo Fisher Scientific). HAECs were washed with TBS and nuclei were stained with DAPI. Coverslips were washed and mounted on slides with an antifade medium. Images were acquired using the same microscope and objective as employed for the migration assays.

### Quantification of actin-based ruffles

Cells were scored positive for lamellipodia/ruffles if there was at least one thick, curled, actin-rich structure per cell, similar to quantification previously described [37]. Only cells with less than one cell border were counted. Cells were quantified as a percentage of the total number of DAPI-stained nuclei. Approximately 50 cells were counted for each experiment and the averages of three different experiments were calculated.

### Statistics

In Table 1, for the longitudinal cohort, continuous variables were analyzed using one-way ANOVA, and for the cross-sectional cohort, the Student’s *t* test was used. The categorical variables were analyzed in the longitudinal cohort using the χ^2^ goodness-of-fit test, and in the cross-sectional cohort using Fisher’s exact test. Data from the PEA assay was analyzed using R, version 3.3.2 [38]. The longitudinal cohort was analyzed using linear mixed models accounting for age, sex, race, matching across BMI groups, and repeated measurements. For EV concentration and size, the differences were analyzed using the Mann–Whitney test. The cell migration and morphology data were analyzed using one-way analysis of variance (ANOVA) and Tukey’s post hoc test.

## Results

### Association of EV protein levels with diabetes status

**Fig. 1:**
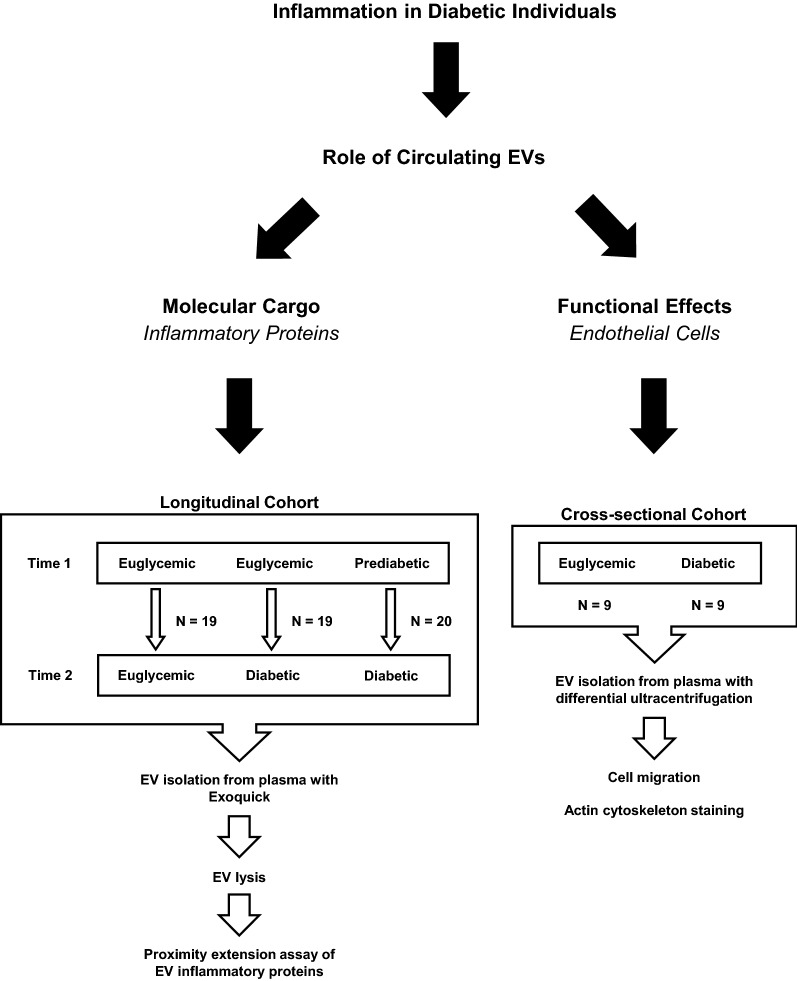
Cohort design. The longitudinal cohort consisted of euglycemic individuals and individuals with prediabetes or diabetes. The cross-sectional cohort consisted of euglycemic and diabetic individuals

Previous data suggested that EVs from diabetic individuals may contribute to heightened inflammation [27]. Hence, we examined whether the inflammatory protein content differed between EVs isolated from diabetic individuals and those of euglycemic controls. Previously, we constructed a longitudinal cohort comprised of individuals who became diabetic over a ~ 5 year time period (4.95 ± 0.23). Individuals were either euglycemic or prediabetic and then became diabetic or they were euglycemic at both time points (Fig. 1). We previously reported that individuals who developed diabetes over this time interval had a higher concentration of plasma EVs than euglycemic controls [27]. Demographic information about this cohort is listed in Table 1 and further characterization of EVs from this cohort was previously described [27]. A flow diagram describing the cohort design is shown in Fig. 1.

In order to analyze the inflammatory protein content of EVs from this cohort, the EVs were lysed and analyzed using a multiplex PEA. This assay quantifies inflammatory proteins in EVs using a sensitive and specific detection method [39–43]. As shown in Table 2, many significant associations were found between the levels of EV inflammatory proteins and the acquisition of diabetes in our longitudinal cohort. Proteins with significant associations included CCL28, CD40, CD5, STAMBP, TWEAK, and VEGF-A, which are indicated in the Table. We also performed a cross-sectional analysis of inflammatory protein levels at time 2 of the longitudinal cohort, where additional significant associations were found between EV inflammatory protein levels and diabetes status. Proteins with significant associations included CD5, MCP-1, and VEGF-A. Some proteins were only significantly associated with one of the diabetes diagnoses groups. However, these groups had different starting conditions (i.e. non-diabetic or pre-diabetic), which may have affected the magnitude of change and the strength of the associations with the EV protein levels.

**Table 2:** Association of EV inflammatory protein levels with diabetes status

Protein	Longitudinal^a^				Longitudinal: time 2^b^		
	*NoDx → NoDx*		HOMA		*NoDx*	HOMA	
	*vs.*				*vs.*		
	*PreDM → DM*	*NoDx → DM*	HOMA-B^c^	HOMA-IR^c^	*DM*	HOMA-B^c^	HOMA-IR^c^
CCL11	0.6895	0.4734	0.0613	0.7986	0.5876	0.1310	0.9978
CCL19	0.0625	0.2538	0.6472	0.2876	0.0704	0.2734	0.0677
CCL23	0.2579	0.2867	0.2974	0.4728	0.9022	0.9583	0.8806
CCL28	0.0008***	0.1180	0.9184	0.8612	0.0514	0.5241	0.4104
CCL4^c^	0.7288	0.1478	0.3385	0.7961	0.1363	0.4471	0.8941
CD40	0.5461	0.0344*	0.3137	0.1580	0.2250	0.8984	0.6521
CD5	0.1318	0.0009***	0.6239	0.3360	0.0324*	0.9095	0.4468
CST5	0.3237	0.5421	0.8888	0.9308	0.9194	0.4713	0.7005
CXCL1	0.9606	0.8884	0.6823	0.5690	0.9593	0.7767	0.4216
CXCL10	0.4053	0.0984	0.1801	0.3023	0.2059	0.1948	0.4656
CXCL11	0.8048	0.7669	0.6952	0.6708	0.3850	0.8246	0.6848
CXCL5	0.7495	0.9368	0.2769	0.7282	0.5621	0.9088	0.7494
CXCL6^c^	0.6071	0.8822	0.3341	0.5976	0.7739	0.5185	0.7089
CXCL9	0.2768	0.6001	0.8169	0.5981	0.9867	0.1415	0.1167
DNER	0.5363	0.2915	0.2429	0.0282*	0.9784	0.0900	0.0090**
FGF-19	0.7586	0.8874	0.3316	0.9467	0.6444	0.3248	0.8076
FGF-21^c^	0.1155	0.6767	0.8559	0.1220	0.1364	0.8983	0.3027
GDNF	0.7608	0.4757	0.8778	0.9899	0.6698	0.2500	0.3030
HGF	0.0947	0.6227	0.3132	0.0127*	0.9682	0.2367	0.0399*
IL-10RB	0.5333	0.3593	0.0226*	0.0407*	0.5623	0.1827	0.2961
IL-18R1	0.9183	0.4255	0.0489*	0.0037**	0.7629	0.2615	0.0172*
LAP TGF-β-1	0.5226	0.8291	0.0949	0.0537	0.7723	0.5630	0.9387
MCP-1	0.0691	0.2371	0.9937	0.5390	0.0409*	0.6267	0.8876
MCP-2	0.2893	0.3501	0.9501	0.4315	0.5721	0.5648	0.9228
MMP-1	0.6481	0.7230	0.8960	0.6673	0.5535	0.3654	0.9796
OPG	0.3266	0.4886	0.7252	0.1477	0.4301	0.5329	0.0611
SCF	0.2646	0.9151	0.0014**	0.2380	0.3614	0.0002***	0.1508
STAMBP	0.8042	0.0043**	0.4552	0.7957	0.1430	0.5963	0.4856
TRAIL	0.3993	0.6607	0.7377	0.8997	0.1053	0.2518	0.3672
TWEAK	0.0180*	0.2933	0.9389	0.6087	0.9182	0.5996	0.2648
uPA	0.2188	0.3251	0.2336	0.1949	0.1293	0.0295*	0.0564
*VEGF-A*	0.0003***	0.0038**	0.0302*	0.0028**	0.0033**	0.6355	0.0477*
4E-BP1	0.3764	0.2595	0.6119	0.4004	0.2777	0.9704	0.4282

EV inflammatory proteins were quantified using PEA. Linear mixed model regression was used to analyze the relationship between EV inflammatory protein levels and diabetes status, HOMA of β-cell function (HOMA-B) and HOMA of insulin resistance (HOMA-IR), with all P-values indicated. Proteins with levels that were significantly associated with any of the variables in the columns are indicated (*** P < 0.001, ** P < 0.01, and * P < 0.05)

^a^Comparison of individuals who developed diabetes mellitus over time versus individuals who were euglycemic at both time points. (*NoDx* euglycemic, *PreDM* prediabetes, DM diabetes mellitus)

^b^Cross-sectional analysis of individuals from the longitudinal cohort who were diagnosed with diabetes mellitus at time 2 versus individuals who were euglycemic at time 2

^c^Natural log-transformed for analysis due to skewness of distribution

Homeostatic model assessment is a mathematical model that measures insulin resistance and β-cell function [34]. Many clinical and epidemiological studies employ the HOMA model to measure the severity of diabetes in individuals [44]. We wanted to examine the various inflammatory proteins in relation to a different quantitative assessment of diabetes mellitus. In our proteomics analysis, we found significant associations between EV inflammatory protein levels and HOMA-B as well as HOMA-IR in our longitudinal analyses and also in our cross-sectional analysis at time 2. According to the longitudinal analysis, proteins with significant associations with HOMA-B included IL-10RB, IL-18R1, SCF, and VEGF-A, and proteins with significant associations with HOMA-IR included DNER, HGF, IL-10RB, IL-18R1, and VEGF-A. In the cross-sectional analysis of the longitudinal cohort at time 2, proteins with significant associations with HOMA-B included SCF and uPA, and proteins with significant associations with HOMA-IR included DNER, HGF, IL-18R1, and VEGF-A.

**Fig. 2:**
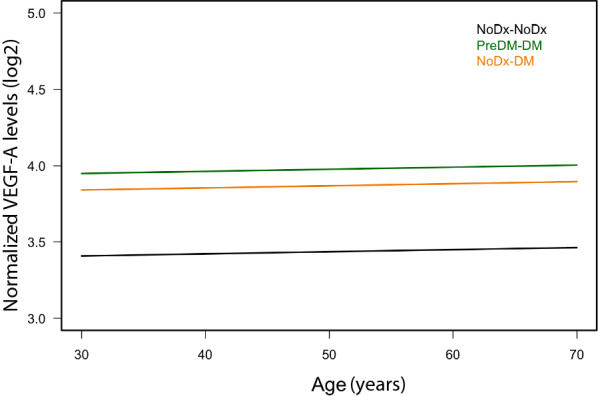
Significant association of EV VEGF-A levels with diabetes mellitus status. EV VEGF-A levels in the longitudinal cohort showed a significant difference between the euglycemic to diabetes and prediabetes to diabetes groups compared to the euglycemic control group (NoDx, euglycemic; PreDM, individuals with prediabetes; DM, individuals with diabetes). The lines represent the predicted values from linear mixed model regression and include two time points for each person, thus time is depicted by age in the graph. Normalized VEGF-A protein levels (log_2_) are shown. The analysis accounted for age, sex, race, matching by BMI groups, and repeated measurements for the longitudinal data. *P *< 0.001 for the PreDM to DM group and *P *< 0.01 for the NoDx to DM group when each is compared to the NoDx to NoDx group

Out of all the examined proteins, EV-associated vascular endothelial growth factor A (VEGF-A) levels showed significant associations with most of the variables analyzed (Table 2). Levels of VEGF-A in EVs were significantly associated with diabetes status in our analyses of the longitudinal cohort at both times and at time 2. Individuals who were prediabetic or euglycemic at time 1 and diagnosed with diabetes at time 2 showed significantly higher levels of VEGF-A in their EVs than individuals who were euglycemic at both time points (Fig. 2). Figure 2 visualizes the predicted values from linear mixed model regression showing that individuals who developed diabetes had higher levels of EV VEGF-A over time. This analysis included two time points for each person, and thus we have depicted time using age in the graph. Figure 2 shows the change in EV VEGF-A levels by age in the three different diabetes diagnosis groups. Furthermore, EV VEGF-A levels were significantly associated with levels of HOMA-B and HOMA-IR in our longitudinal analysis, and HOMA-IR in our cross-sectional analysis at time 2. VEGF-A is a widely known angiogenic factor and its binding to receptors, including VEGFR-1 and VEGFR-2, regulates angiogenesis [45]. VEGF-A plays an important role in the formation of vasculature by stimulating a variety of processes, including endothelial cell migration [46].

### EVs from individuals with diabetes increase cell migration

Given the association that we found between EV inflammatory protein content and diabetes status, we examined whether EVs from diabetic individuals affected cell behavior in vitro. Diabetes is known to be associated with an increased risk for vascular disease, which may arise from interactions between circulating EVs and the endothelium [31]. Hence, we used endothelial cells to see whether EVs from diabetic individuals can induce functional effects.

**Fig. 3:**
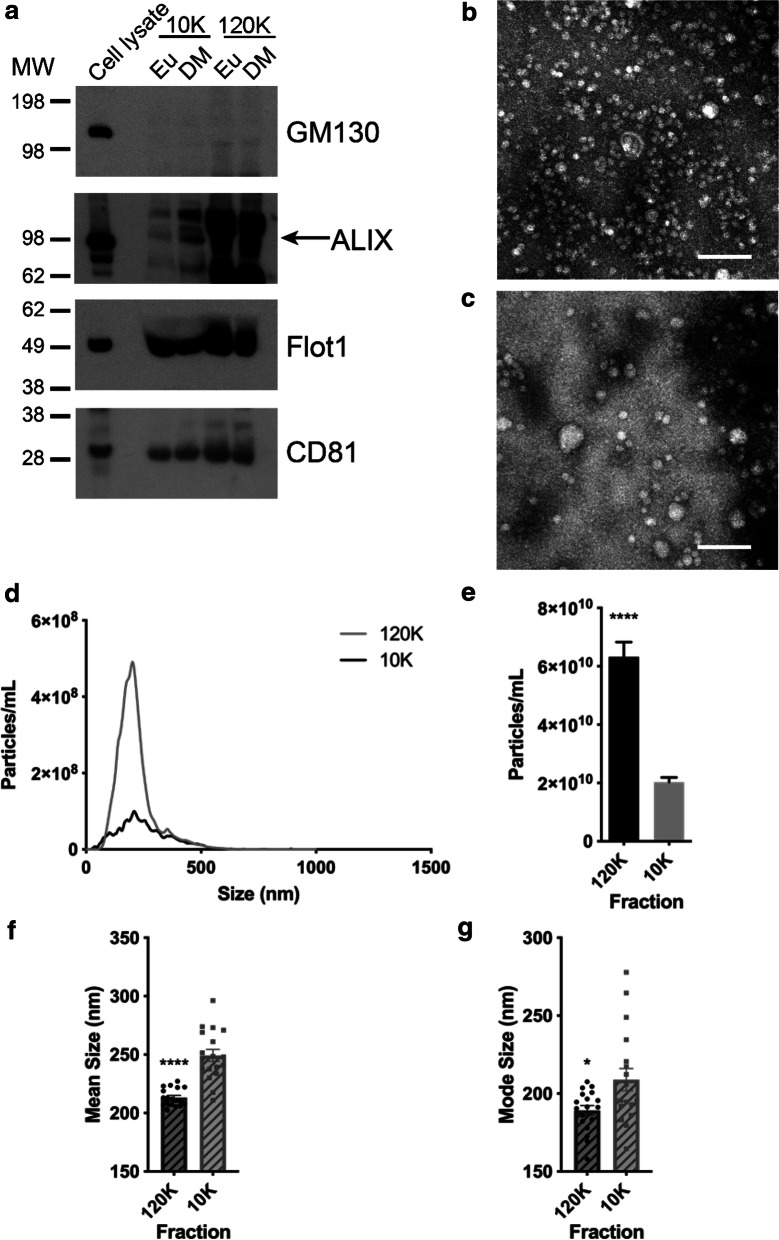
Characterization of EVs from euglycemic and diabetic individuals. **a** Cell lysate and plasma EV samples, isolated from differential ultracentrifugation, were lysed with M-PER and analyzed with SDS PAGE followed by probing for known EV markers, including Alix, Flotillin 1, and CD81, in addition to probing for the EV purity marker, GM130. **b** Electron microscopy of EVs from plasma, isolated with the 120K fraction of differential ultracentrifugation, exhibit expected EV morphology and size. Scale bar = 200 nm. **c** Electron microscopy of EVs from plasma, isolated with the 10K fraction of differential ultracentrifugation, exhibit expected EV morphology and size. Scale bar = 200 nm. **d**, **e** EVs were isolated from the cross-sectional diabetes cohort. Size distributions and concentrations from NTA analysis were averaged for both the 120K and 10K groups, each including data from 18 individuals. The area under the curve for each group in D are reflected in the corresponding average concentration shown in the histogram in **e**. **f** EV mean and (**g**) mode size from NTA analysis were averaged for both the 120K and 10K groups. In each graph, dots indicate data from 18 individuals. Histograms represent the average ± SEM. ****P < 0.0001 and *P < 0.05 using a Mann–Whitney test in **e**–**g**

For these studies, plasma EVs from the cross-sectional cohort were isolated using differential ultracentrifugation (Fig. 1). We chose to use this isolation method since precipitation reagents contain polyethylene glycol, which may confound the outcomes of cell migration assays [47, 48]. Guidelines from the International Society for Extracellular Vesicles were followed in characterizing and validating the presence of isolated EVs [49]. EVs were isolated based on size via differential ultracentrifugation, in which the 10,000 *g* (10K) and 120,000 *g* (120K) fractions contain, respectively, medium-sized and sEVs [23]. The 10K and 120K fractions showed signal for known EV markers and no signal for the EV purity marker (Fig. 3a). Images taken with electron microscopy show intact, round vesicles for both the 10K and 120K samples (Fig. 3b, c). The size distributions for the 10K and 120K fractions were quantified using NTA (Fig. 3d). The EVs from the 120K fraction have a typical size distribution of plasma EVs and show a peak at approximately 200 nm. In comparison, EVs from the 10K fraction have a broader distribution and a significantly lower concentration of particles (Fig. 3d, e). Comparison of EV mean and mode sizes indicate that EVs isolated from the 10K fraction were significantly larger than EVs isolated from the 120K fraction (Fig. 3f, g). Hence, our samples isolated from differential ultracentrifugation exhibit size and morphological characteristics of EVs.

**Fig. 4:**
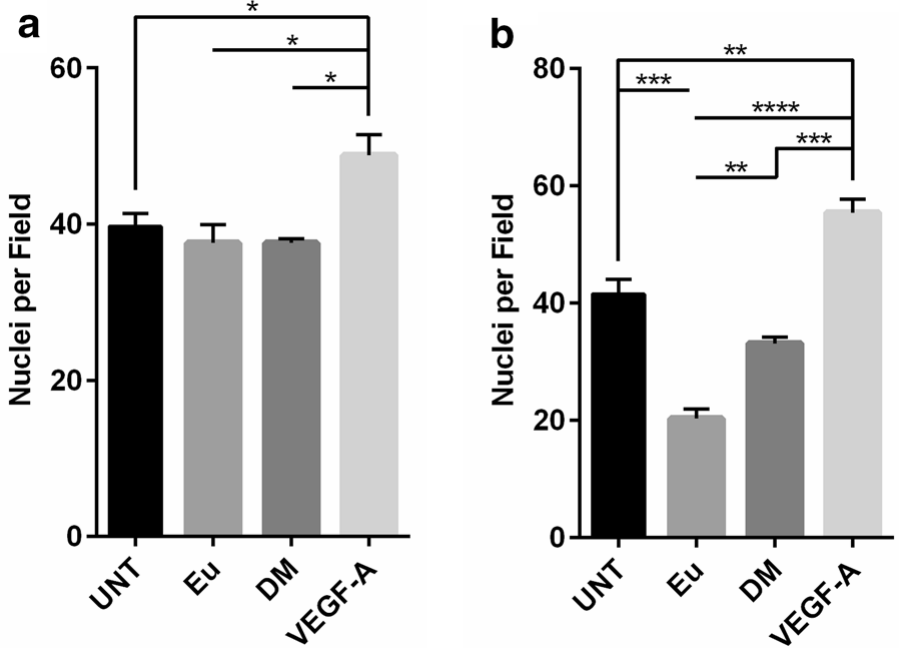
Medium-sized EVs from individuals with diabetes enhanced cell migration in comparison to those from euglycemic individuals. HAECs were allowed to migrate for 3 h toward collagen-coated inserts. The bottom well contained VEGF-A as a positive control or euglycemic and diabetic sEVs (120K fraction) at a dose of 1.8 × 10^8^ vesicles, with each well containing EVs equally pooled from three individuals (**a**). Migration was also tested using medium-sized EVs (10K fraction) at the same dose and pooling size (**b**). PBS was used as a negative control for cells that were untreated (UNT) with EVs. Histograms represent the mean number of migrated cells in triplicate wells ± SEM. ***P < 0.001, **P < 0.01, and *P < 0.05 for the indicated comparisons using one-way ANOVA and Tukey’s post hoc test

Based on our proteomic analysis showing that EV inflammatory protein levels are significantly associated with diabetes status, we wanted to see if EVs that differed in molecular cargo also differed in their functional effects. Using the Boyden transwell assay, we allowed HAECs to migrate towards either EVs, isolated from individuals in our cross-sectional cohort, or VEGF-A. EVs from the 120K and 10K spins of differential ultracentrifugation were classified as, respectively, sEVs and medium-sized EVs [23]. Our results showed no differences in endothelial cell migration towards sEVs (120K fraction) isolated from euglycemic or diabetic individuals, with the migration from both groups being similar to that of the untreated endothelial cells (Fig. 4a). In contrast, our subsequent test with medium-sized EVs (10K fraction) showed greater cell migration with EVs derived from diabetic individuals than compared to the euglycemic control (Fig. 4b).

### EVs in diabetes alter cell morphology

**Fig. 5:**
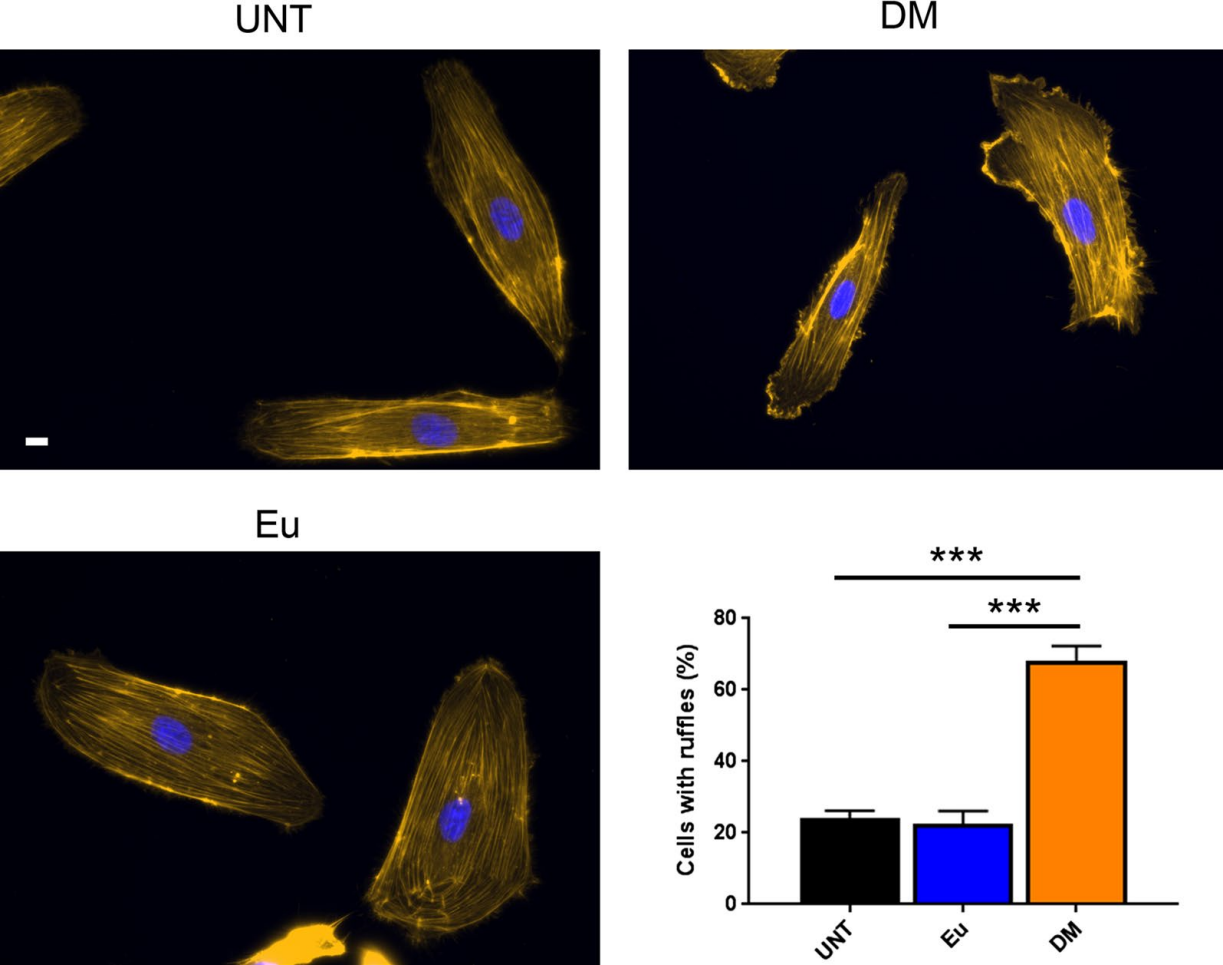
Medium-sized EVs from individuals with diabetes induced lamellipodia formation in endothelial cells. HAECs were either untreated or treated with EVs from the 10K fraction at a dose of ~ 1000 vesicles per cell for 24 h and then stained with Rhodamine Phalloidin and DAPI. Cells treated with EVs from a diabetic individual displayed significantly more actin-rich, membrane protrusions and ruffles compared to untreated cells or cells treated with EVs from the euglycemic control. Lamellipodia were quantified and expressed as a percentage of the total number of DAPI-stained cells. The histogram represents the average number of cells from three replicate experiments ± SEM. ***P < 0.001 for the indicated comparisons using one-way ANOVA and Tukey’s post hoc test

Since endothelial cell migration is mobilized by the actin cytoskeleton [50], we tested whether medium-sized EVs (10K fraction) from individuals with diabetes may lead to structural alterations in target cells. After incubating HAECs with EVs from diabetic or euglycemic individuals, we stained the actin cytoskeleton of the cells. Our results show that HAECs displayed significantly more actin-rich, membrane protrusions and ruffles, called lamellipodia, when treated with EVs from diabetic rather than euglycemic individuals, and that EVs from the latter led to morphology similar to what was seen in the untreated cells (Fig. 5). There were no significant changes in cell size across all categories (Additional file 1: Figure S1).

## Discussion

Our previous work on a human cohort showed that diabetic individuals have increased levels of plasma EVs and suggested that EVs may be involved in the inflammatory pathways of diabetes mellitus [27]. In this study, we examined the inflammatory cargo of EVs from diabetic individuals. Through analyses of our longitudinal cohort, we found significant associations between EV inflammatory protein levels and diabetes status. Furthermore, we found that EVs from diabetic individuals altered the migration and morphology of human aortic endothelial cells when compared to euglycemic controls.

Inflammation is a contributor to endothelial dysfunction [16], which leads to the development of vascular complications in diabetes. Hence, we attempted to elucidate the role of EVs in the progression of these comorbidities by studying EV-associated factors that are involved in inflammation. Previous research on EVs in diabetic inflammation have examined EVs from adipose tissue and characterized microRNA content within these vesicles [25, 51]. Our work in this study focused on EVs derived from the circulation of diabetic individuals and profiling the EV protein content by testing a large panel of inflammatory biomarkers.

Notably, EV VEGF-A levels were associated with diabetes status, HOMA-B and HOMA-IR levels in the longitudinal cohort, as well as in our cross-sectional analysis of diabetes status and HOMA-IR levels at time 2. Our results corroborate with a previous cross-sectional study that observed higher levels of VEGF in platelet-poor plasma EVs from diabetic individuals [52]. Our longitudinal study expands upon those findings by suggesting that individuals who develop diabetes over time will acquire higher levels of VEGF-A in their circulating EVs.

Here, we report that EVs from diabetic individuals increased the migration of endothelial cells when compared to EVs from euglycemic individuals. Given the link between cell migration and VEGF content, our findings would be consistent with studies also examining EVs and cell migration but looking at the effects of EV VEGF-A levels on angiogenesis in cancer. Numerous studies have shown that the isolation of EVs-containing VEGF from cancer cells promotes endothelial cell migration and angiogenesis in vitro [53–57]. These studies point to VEGF in EVs as a potent inducer of tumor angiogenesis.

VEGF-A content in EVs is important due to the strong connection between VEGF-A levels and the potential severity of an individual’s diabetes. In a clinical study of euglycemic and diabetic individuals, researchers showed that levels of hemoglobin A1c (HbA1c) were positively correlated with plasma VEGF levels [58]. In agreement with that finding, another study found that concentrations of VEGF-A and receptors 1 and 2 levels were similar in serum samples from patients with well-controlled diabetes and healthy individuals [59]. This result is significant as the individuals with well-controlled diabetes displayed no signs of vascular-related complications. Hence, quantifying VEGF-A in EVs may be a valuable tool in measuring the progression of diabetes. Our cohort study offers such information, in addition to data on a variety of other inflammatory biomarkers.

However, it is also important to note the complex relationship between VEGF levels and the progression of diabetes mellitus. Plasma VEGF levels may not always reflect intracellular organ levels of VEGF [60]. Furthermore, employing anti-VEGF interventions in response to increased VEGF levels may interfere with essential processes throughout the body, including wound healing and collateral vessel development [60]. Hence, before EVs can be deemed as a robust biomarker and therapeutic target for diabetes, it would be necessary to further explore the role of EVs in shuttling VEGF.

Our data indicate that EVs from individuals with diabetes contain cargo that affect cell morphology and migration. In the Transwell Boyden assays, we observed that medium-sized EVs (10K fraction) from diabetic individuals enhanced cell migration when compared to cells incubated with EVs from euglycemic individuals. Hence, our finding suggests that EVs from diabetic individuals carry greater chemoattractant than EVs from euglycemic individuals. The implication of our findings agrees with another report showing higher levels of VEGF in medium-sized EVs from individuals with diabetes, in which the EVs were isolated from the 10K spin of differential ultracentrifugation [52]. Given that VEGF-A levels are more abundant in the EVs of diabetic individuals, we expected the chemoattractant of those EVs to induce proangiogenic behavior in target cells. The hypothesis is in line with immunofluorescence experiments showing that endothelial cells incubated with medium-sized EVs (10K fraction) in diabetes displayed more actin-rich, membrane protrusions and ruffles than compared to cells incubated with EVs from the euglycemic control. Lamellipodia are known to enhance cell migration through actin polymerization and reorganization, which drive the protrusions of the leading edges of cells [50]. Hence, our results suggest that EVs from diabetic individuals carry cargo that promote endothelial cell migration, and thereby, angiogenesis.

The results from the proteomic and in vitro studies are consistent with the excessive angiogenesis that occurs in certain diabetic comorbidities, including diabetic retinopathy [61]. Conversely, our findings may also reflect a compensatory mechanism for comorbidities involving deficient angiogenesis and endothelial dysfunction, including heart disease and stroke [61]. The role of angiogenic factors in vascular disease and diabetes is multi-dimensional and requires further research, as current attempts at angiogenic therapies have drawbacks, including the inability to make targeted deliveries [62]. Those issues could potentially be addressed by engineering EVs to serve as novel carriers of angiogenic and anti-angiogenic factors to selected organs in the body.

In addition to VEGF-A, we found multiple EV inflammatory proteins that may give further insight on the pathophysiology of diabetes, including cluster of differentiation 40 (CD40). The interaction between CD40 and its corresponding ligand, CD40L, is known to induce platelet activation, thereby leading to inflammation and atherosclerosis [63]. In a study of humans who had no history of vascular complications, when compared to controls, individuals with type 2 diabetes had higher levels of soluble CD40L, which have been associated with an increased risk for cardiovascular events [64]. In our longitudinal study, EV levels of CD40 are significantly associated with the status of individuals who transitioned from a euglycemic to diabetic diagnosis. Thus, CD40 content in EVs may help to predict vascular disease for diabetes mellitus.

Hepatocyte growth factor (HGF) is another EV-associated inflammatory protein that may be relevant to vascular disease. Levels of HGF in EVs are significantly associated with HOMA-IR levels in our longitudinal analysis of the cohort and in our cross-sectional analysis of time 2. It has been reported that HGF may have protective effects against insulin resistance by allowing for regeneration of β cells in diabetes mellitus [65]. Furthermore, HGF has been found to modulate anti-inflammatory responses in murine models [65]. Hence, examining EV HGF content may provide more knowledge on the biological mechanisms that counteract insulin resistance and elucidate the complex role EVs play in individuals with diabetes.

Similar to HGF, EV levels of IL-18R1, a receptor for IL-18, was found to be significantly associated with HOMA-IR in our longitudinal analysis and in our cross-sectional analysis of time 2. It has been found that plasma IL-18 levels are significantly associated with HOMA-IR, and this relationship is independent of obesity and diabetes status [66]. In turn, EVs may serve as a useful assessment of insulin resistance in diabetes mellitus.

Here, we have analyzed levels of EV inflammatory proteins. Many cytokines can be encapsulated in EVs and have been shown to be biologically active [67]. Intriguingly, cytokines in the soluble versus EV-encapsulated fraction differ and may depend on the biological system and stimulus [67]. It would be interesting in the future to examine the inflammatory profiles of the soluble along with EV-associated fraction. This avenue of exploration would build upon previous literature showing that microRNA profiles in the EV-enriched serum fraction differed between individuals with normal glucose tolerance and diabetes, but the microRNAs did not differ in the soluble fraction of serum [68]. Therefore, biological cargo may be sorted differentially into the soluble versus EV-associated fractions in type 2 diabetes mellitus.

Lastly, results from our functional assays show that the 10K fraction, containing medium-sized EVs, caused alterations in recipient endothelial cells. It has only recently been appreciated that EVs from the 10K and 100K fractions may carry different molecular cargo and, hence, may elicit different biological functions [23]. In general, most studies have focused on one fraction of EVs when examining effects on endothelial cells. In fact, many previous studies omitted the 10K step of differential ultracentrifugation and thus collected both medium-sized and sEVs [56, 69, 70]. sEVs obtained from the 100K fraction have been demonstrated to elicit effects on endothelial cell function [55, 57, 71, 72, 73]. Other EV isolation techniques, that non-discriminately collect for both small and medium-sized EVs, have also been done for assays studying endothelial cell migration [53]. Here, our findings contribute to the more limited literature on the role of EVs isolated from the 10 K fraction of differential ultracentrifugation on endothelial cell migration and angiogenesis [74]. The role of medium-sized EVs in endothelial cell migration may be connected to the described importance of these vesicles in mediating endothelial dysfunction and cardiovascular disease [75]. We have evidence pointing towards the proangiogenic effects of medium-sized EVs (10 K fraction), which may contribute to vascular complications in diabetes. Hence, these results may further our understanding of the properties and functions that differentiate medium-sized EVs from other types of EVs within the context of pathological conditions.

## Conclusions

Our results demonstrate that EV inflammatory protein profiles differ by diabetes status. We also have preliminary evidence suggesting that the inflammatory protein cargo in EVs from diabetic individuals can functionally alter endothelial cells, including changes to cell morphology and migratory behavior. Follow-up studies using tissue from targeted organs will likely contribute to a better understanding of how EVs can compromise or promote vascular health. In turn, EVs may become a valuable diagnostic tool for diabetes mellitus, which would enable us to address an alarming epidemic and assist disproportionately impacted populations.

## Supplementary information

**Additional file 1: Figure S1** Cell area of endothelial cells treated with EVs from euglycemic or diabetic individuals.

## Data Availability

The datasets generated and analyzed during the current study are available from the corresponding author upon reasonable request through the HANDLS website, https://handls.nih.gov/.

## Abbreviations

EV: Extracellular vesicle
VEGF: vascular endothelial growth factor
HOMA: Homeostatic model assessment
IL-18R1: Interleukin-18 receptor 1
IR: Insulin resistance
B: β-Cell function
CVD: Cardiovascular disease
sEV: Small extracellular vesicle
HANDLS: Healthy Aging in Neighborhoods of Diversity across the Life Span
NIA: National Institute on Aging
NIH: National Institutes of Health
BMI: Body mass index
PBS: Phosphate-buffered saline
HEPES: 4-(2-Hydroxyethyl)piperazine-1-ethanesulfonic acid
M-PER: Mammalian Protein Extraction Reagent
NTA: Nanoparticle tracking analysis
PEA: Proximity Extension Assay
ANOVA: Analysis of variance
HAEC: Human aortic endothelial cell
DAPI: 4′,6-Diamidino-2-phenylindole
CD40: Cluster of differentiation 40
HGF: Hepatocyte growth factor
