# Supplementary material for: Extracellular vesicles in diabetes mellitus induce alterations in endothelial cell morphology and migration
Source: J Transl Med. 2020 Jun 9;18:230. doi: 10.1186/s12967-020-02398-6 (PMC7285586; doi:10.1186/s12967-020-02398-6)
Supplement: Supplementary file 1 — Additional file 1: Figure S1 Cell area of endothelial cells treated with EVs from euglycemic or diabetic individuals. [file 12967_2020_2398_MOESM1_ESM.pdf]

<u>Additional file 1: Fig. S1</u>

Extracellular vesicles in diabetes mellitus induce alterations in endothelial cell morphology and migration

Sharon F. Wu, Nicole Noren Hooten, David W. Freeman, Nicolle A. Mode, Alan B. Zonderman, and Michele K. Evans

Additional methods

Quantification of cell area

Borders of the cells were traced using ImageJ and the areas were measured in arbitrary units. Only cells that had less than one cell border were counted. Approximately 90 cells were counted for each experiment and the average areas of the three different experiments were calculated (Fig. S1).

Fig. S1

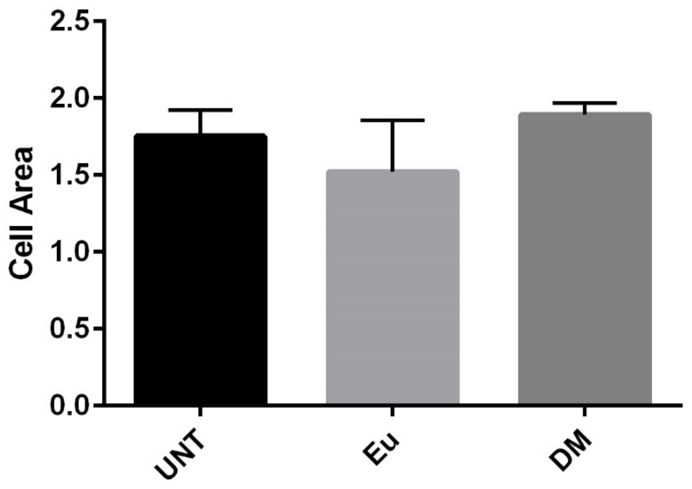

Fig. S1 Cell area of endothelial cells treated with EVs from euglycemic or diabetic individuals. Endothelial cells that were either untreated (UNT) or treated with medium-sized EVs from euglycemic or diabetic individuals showed similar cell areas in the immunofluorescence experiments. The histogram represents the average area of cells from three replicate experiments ± SEM. No statistically significant differences between the categories were found from comparisons using one-way ANOVA and Tukey's post-hoc test.
